# Fortification of Chinese Steamed Bread Through Broken *Ganoderma lucidum* Spore Powder Incorporation: Effects on Physicochemical and Quality Properties

**DOI:** 10.3390/foods14081433

**Published:** 2025-04-21

**Authors:** Jia Chen, Deyu Cheng, Siyi Luo, Yilan Hu, Chun Liu, Xingfeng Guo, Xiuzhu Yu, Lingyan Zhang, Jihong Wu

**Affiliations:** 1 School of Pharmaceutical Sciences and Food Engineering, Liaocheng University, 1 Hunan Road, Liaocheng 252000, China; jiachenlcu@163.com (J.C.); 19563390693@163.com (D.C.); sy2983143728@163.com (S.L.); hushasha2025@163.com (Y.H.); 19506803086@163.com (C.L.); guoxf0906@126.com (X.G.); 2 Shandong Key Laboratory of Applied Technology for Protein and Peptide Drugs, Liaocheng University, 1 Hunan Road, Liaocheng 252000, China; 3 Engineering Research Center of Grain and Oil Functionalized Processing in Universities of Shaanxi Province, College of Food Science and Engineering, Northwest A&F University, 22 Xinong Road, Yangling, Xianyang 712100, China; xiuzhuyu@nwafu.edu.cn; 4 College of Food Science and Nutritional Engineering, China Agricultural University, 17 Tsing Hua East Road, Beijing 100083, China

**Keywords:** Chinese steamed bread, *Ganoderma lucidum* spore powder, specific volume, texture, flavor

## Abstract

Broken *Ganoderma lucidum* spore powder (BGLSP) is abundant in nutrients and bioactive compounds, rendering it a suitable functional raw material for food applications. This study examined the impact of incorporating BGLSP (ranging from 0.5% to 10%) on the physicochemical properties of flour blends, dough, and the quality of Chinese steamed bread (CSB). The results indicated that with increasing BGLSP content, the a* value, onset temperature, peak temperature, water absorption, development time, and dough stability all exhibited an upward trend in the flour blends and dough, while the L* value and protein network weakening decreased. When compared to the control sample, the inclusion of 10% BGLSP resulted in a reduction in the spread ratio, specific volume, cohesiveness, and springiness of CSB, while simultaneously increasing its hardness, chewiness, and gumminess. The observed odor variations among samples were primarily ascribed to the proportions of aldehydes and ketones. Notably, sensory evaluation demonstrated that the flavor attributes of BGLSP-enhanced samples were superior to those of the control sample. In conclusion, the incorporation of BGLSP at concentrations ranging from 0.5% to 1% is deemed optimal for CSB, offering novel insights into the application of BGLSP within the food industry.

## 1. Introduction

Chinese steamed bread (CSB), commonly referred to as *Mantou*, has served as a staple food in China since ancient times. It is a traditional fermented and steamed product primarily composed of refined wheat flour, water, and yeast/sourdough [1]. Refined wheat flour is notably deficient in dietary fiber, containing less than 0.6%, due to the loss incurred during processing. This deficiency is associated with an elevated risk of cardiovascular diseases, diabetes, obesity, as well as gastrointestinal and degenerative disorders [2]. Consequently, there has been a growing demand for staple food products that are enriched with dietary fibers.

*Ganoderma lucidum* (*G. lucidum*) has historically been regarded as a medicinal and culinary fungus in ancient China, reputed for its enigmatic therapeutic effects on both the body and mind, contributing to longevity [3]. The composition of the substance primarily includes ash (0.72–1.77%), carbohydrates (21.83–27.78%), fats (1.1–8.3%), fibers (59–65%), proteins (7–8%), among other components [4]. The species contains over 400 bioactive compounds, such as polysaccharides, triterpenoids, and glycopeptides, which have been extensively utilized in the prevention and treatment of conditions including neurasthenia, cancer, and arthritis, and for detoxification purposes [5,6]. Research has shown that the incorporation of dietary fibers, polyphenols, and proteins positively influences the textural, antioxidant, and digestive properties of CSB [1,2]. The extract of *G. lucidum* has been processed into various forms, such as tea, powder, and dietary supplements, which are commercially available for the treatment of various ailments [7]. While the fruiting body of *G. lucidum* has long been employed in traditional herbal medicine, its spores also possess a substantial quantity of components similar to those found in the fruiting body. Furthermore, the activity of the spores is closely linked to the condition of the sporoderm. When the sporoderm remains intact, the pharmacological effects are diminished; conversely, breaking the sporoderm enhances the release of active components and the exertion of their effects. Current research on *G. lucidum* spores primarily focuses on extraction and separation, the analysis of biological constituents, and pharmacological activities [8,9]. However, the effects of *G. lucidum* spores on dough and CSB quality have not been investigated, and there is a notable gap in the literature regarding their application in staple food products.

**Figure 1:**
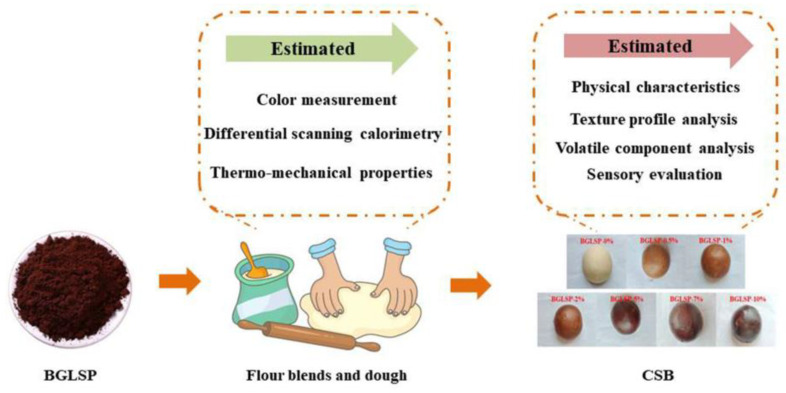
Diagrammatic representation of experimental design. Abbreviations: BGLSP, broken *G. lucidum* spore powder; CSB, Chinese steamed bread.

This study employed broken *G. lucidum* spore powder (BGLSP) at various concentrations as a functional ingredient in CSB. The investigation focused on the impact of BGLSP on the physicochemical properties, including color, thermal properties, and thermo-mechanical properties, of flour blends and dough, as well as the quality characteristics, such as moisture content, spread ratio, specific volume, texture, sensory evaluation, and volatile components, of the resulting CSB (Figure 1). The findings of this research may encourage further exploration into the application of BGLSP in the development of innovative products.

## 2. Materials and Methods

### 2.1. Materials

Wheat flour (Yihai Kerry Co., Ltd., Qingdao, China) and commercial instant dry yeast (Angel Yeast Co., Ltd., Yichang, China) were sourced from a local supermarket in Liaocheng, while BGLSP was procured from Anhui Guzhitang Pharmaceutical Co., Ltd. (Haozhou, China).

### 2.2. Preparation of Flour Blends and CSB

**Figure 2:**
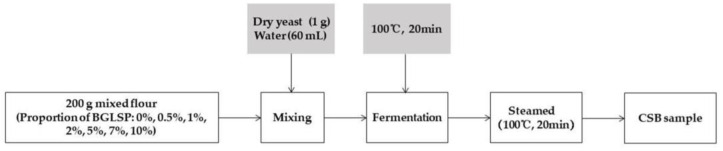
The main procedure for production of CSB with BGLSP added. Abbreviations: BGLSP, broken *G. lucidum* spore powder; CSB, Chinese steamed bread.

The preparation of CSB followed the methodology outlined by Gao et al. [10], with minor modifications (Figure 2). The control CSB formulation comprised 200 g of wheat flour, 1 g of dry yeast, and 60 mL of distilled water. Based on the preliminary experimental results, and considering the color and softness of the products, the addition of BGLSP should not exceed 10%. Thus, CSBs were produced using wheat flour partially substituted with BGLSP at levels of 0.5%, 1%, 2%, 5%, 7%, and 10%. All ingredients were combined using a dough mixer (Ashton Electric Co., Ltd., Ningbo, Zhejiang, China) operating at a low speed of 50 rpm for a duration of 5 min to achieve a homogenous dough consistency. Following the mixing process, the dough underwent fermentation at 35 °C with a relative humidity of 85% for 90 min within a controlled fermentation chamber. Subsequently, the fermented dough was subjected to steaming in boiling water for 20 min and then allowed to cool completely at ambient room temperature for 1 h. The quality of the CSB was evaluated in subsequent experimental analyses.

### 2.3. Color Measurement of Flour Blends

The color parameters of each sample were assessed utilizing a colorimeter (CR-400 Chroma Meter, Konica Minolta Sensing Inc., Osaka, Japan), with measurements recorded for lightness (L*), redness (a*), and yellowness (b*). Before measurement, the colorimeter was calibrated using a standard white plate. The total color difference (Δ*E*) was calculated using the following equation to evaluate the overall color change relative to the control. The parameter Δ*E* effectively quantifies the extent of color variation between wheat flour and flour blends.

(1) $$∆E={\left[{\left(L_{0}^{*}-L^{*}\right)}^{2}+{\left(a_{0}^{*}-a^{*}\right)}^{2}+{\left(b_{0}^{*}-b^{*}\right)}^{2}\right]}^{1/2}$$

where *L*_0_*, *a*_0_*, and *b*_0_* are the control values.

### 2.4. Differential Scanning Calorimetry (DSC) Analysis of Flour Blends

The thermal properties of the flour blends were analyzed employing a NETZSCH DSC 204 F1 differential scanning calorimeter (Netzsch, Selb, Germany), following the methodology outlined by Menegassi et al. [11], with certain modifications. In brief, approximately 2 mg of each sample was weighed into 40 mL aluminum pans. Distilled water was added via pipette to achieve a water-to-sample ratio of 3:1 in the DSC pans. The pans were sealed securely and left to equilibrate for 12 h prior to analysis. Measurements were conducted over a temperature range of 30 to 120 °C, at a heating rate of 10 °C/min, under an air atmosphere. Parameters such as onset temperature (*T*_o_), peak temperature (*T*_p_), conclusion temperature (*T*_c_), and enthalpy change (Δ*H*) were determined.

### 2.5. Thermo-Mechanical Properties of Dough

Following the methodology outlined by Wang et al. [12], the impact of BGLSP on the thermo-mechanical properties of dough was assessed using a Mixolab 2 (Chopin Technologies, Tripette et Renaud, Paris, France). The rheological behavior was analyzed under the following Mixolab procedure parameters: a tank temperature of 30 °C, a mixing speed of 80 rpm, a target torque of 1.1 Nm (±0.05 Nm), a total dough mass (comprising flour and water) of 75 g, and an analysis duration of 30 min. Key metrics such as water absorption, dough development time, dough stability time, initial consistency (C1), and protein network weakening (C2) were evaluated. Each sample test was performed in triplicates.

### 2.6. Determination of Physical Characteristics of CSB

The moisture content of CSB was assessed using an oven drying method (105 ± 2 °C), as specified in GB 5009.3-2016. The spread ratio was calculated as the ratio of the diameter to the height of the CSB, following the methodology outlined by Wu et al. [13]. The volume of each CSB sample was determined through millet displacement, as described by Li et al. [14]. The weight of the CSB was measured with an accuracy of 0.01 g. The specific volume of the CSB was expressed as the ratio of volume to weight (mL/g). Each sample underwent triplicate measurement.

### 2.7. Texture Profile Analysis of CSB

For texture determination, the CSB samples were sliced into 5 cm square pieces with a thickness of about 2.5 cm. The texture quality was evaluated using a CT3 Texture Analyzer (Brookfield, Middleboro, MA, USA), based on the method of Feng et al. [15], with minor modifications. The texture analyzer parameters were set as follows: P/25 probe, 50% compression ratio, 500 N testing force, 1 mm/s testing speed, 5 s interval time, and a trigger force of 5 g. The pre-speed, test-speed, and post-speed were set as 2.0, 5.0, and 5.0 mm/s, respectively. Measurements of hardness, chewiness, gumminess, springiness, and cohesiveness were recorded.

### 2.8. Volatile Component Analysis of CSB

The volatile compounds present in CSB were analyzed utilizing a headspace solid-phase microextraction combined with gas chromatography–mass spectrometry (HS-SPME/GC–MS) system, employing a 50/30 μm PDMS/DVB/CAR fiber from Supelco (Bellefonte, PA, USA), as per the methodology previously described by Chen et al. [16]. The GC–MS analysis was conducted using a Shimadzu QP2010 instrument (Shimadzu Corporation, Kyoto, Japan) equipped with a DB-17MS chromatographic column (60 m × 0.25 mm × 0.25 μm, Agilent Technologies, Santa Clara, CA, USA). The SPME fiber was introduced into the GC injector, and the adsorbed volatiles were thermally desorbed at 250 °C for a duration of 3 min. A sample of approximately 5 g of CSB was placed in a 20 mL headspace vial for analysis. The initial oven temperature was set at 40 °C, increased to 120 °C at a rate of 4 °C/min, then further elevated to 240 °C at a rate of 6 °C/min, and maintained at this temperature for 9 min. The identification of volatile compounds was achieved through the use of retention indices and mass spectra, with only those compounds demonstrating a similarity of greater than 85%, with the NIST14 library being considered.

### 2.9. Sensory Evaluation of CSB

The sensory quality of CSBs was assessed using the scoring system outlined in the Chinese Standard GB/T 35991-2018. The evaluation was conducted by a panel of 10 healthy assessors, comprising 5 males and 5 females aged between 22 and 31 years, under consistent environmental conditions. As shown in Table 1, the sensory analysis focused on several attributes, specific volume (maximum score of 20), appearance (maximum score of 15), color (maximum score of 10), internal structure (maximum score of 15), toughness (maximum score of 20), chewing performance (maximum score of 15), and flavor (maximum score of 5), employing descriptive sensory analysis as described by Liu et al. [1].

### 2.10. Statistical Analysis

The data were reported as means ± standard deviations (SDs) based on a minimum of three independent measurements. Statistical comparisons were conducted using one-way ANOVA and Duncan’s multiple range tests, facilitated by SPSS software version 11.5 (SPSS, Inc., Chicago, IL, USA).

## 3. Results and Discussion

### 3.1. Effects of BGLSP on Color of Flour Blends

**Figure 3:**
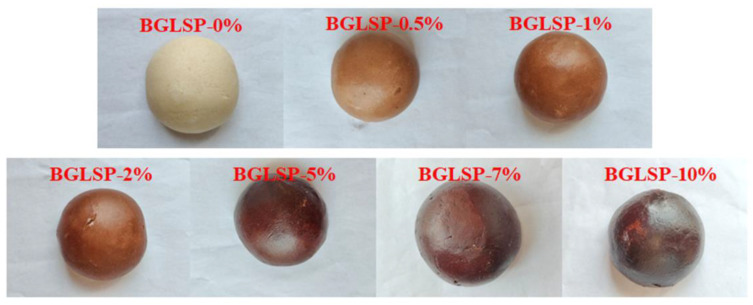
Appearance of CSB. Abbreviations: BGLSP, broken *G. lucidum* spore powder; CSB, Chinese steamed bread.

Color is a crucial attribute affecting the quality of flour blends, significantly impacting the visual appeal of CSB [17]. Table 2 illustrates the impact of BGLSP on the color parameters of flour blends. The yellowness (b*) values initially decreased and then increased with the rise in BGLSP content. The color of CSB primarily originates from the inherent pigmentation of BGLSP, which is additionally influenced by oxidation reactions that may transpire during the steaming process [7,10]. With the incremental addition of BGLSP, there was a statistically significant increase (*p* < 0.05) in the redness (a*) values of the flour blends. Conversely, the lightness (L*) values exhibited a significant decrease (*p* < 0.05) with the incorporation of BGLSP. The Δ*E* value quantifies the extent of color deviation from standard wheat flour, with Δ*E* > 3 denoting a perceptible color difference to the human eye [18]. The Δ*E* values for flour blends containing BGLSP exceeded 3, indicating a marked color difference from the control group. Figure 3 presents the appearance and scanning results of CSB, demonstrating a trend towards darker coloration with increased BGLSP content. This darkening effect is likely attributable to the inherent dark brown hue of BGLSP, which influences the overall color of the CSB [10].

### 3.2. Effects of BGLSP on Thermal Properties of Flour Blends

Upon heating an aqueous suspension of starch granules beyond a specific temperature threshold, the granules experience an irreversible order–disorder transition, termed gelatinization [19]. DSC was employed to characterize the gelatinization process in various flour blends. The gelatinization parameters for these blends are detailed in Table 3. The transition temperatures, namely *T*_o_, *T*_p_, and *T*_c_, along with the gelatinization enthalpy (Δ*H*), exhibited minor variations across the samples. Specifically, *T*_o_ ranged from 56.85 °C to 57.59 °C, *T*_p_ from 62.43 °C to 63.29 °C, and *T*_c_ from 67.39 °C to 68.71 °C. The Δ*H* values for the flour blends varied between 5.86 J/g and 7.24 J/g. Notably, an increase in the BGLSP content corresponded to elevated *T*_o_ and *T*_p_ gelatinization temperatures. The observed variations in gelatinization temperatures and gelatinization enthalpy can be attributed to the differences in lipid, protein, and fiber content among the flour blends [4]. This is due to the fact that the flour blends are not composed of pure starch, allowing for interactions between starch and these additional components. Elevated gelatinization temperatures indicate that starch granules necessitate greater energy input for swelling, thereby influencing dough hydration and stability during the steaming process [20]. Wang et al. [21] demonstrated that flours exhibiting lower gelatinization temperatures resulted in CSB with softer textures, attributable to the rapid swelling of starch, whereas flours with higher gelatinization temperatures produced denser structural characteristics. The observed variation in the values of Δ*H* can be attributed to the differing granulometries, which contain varying amounts of BGLSP. These components may diminish the availability of starch, thereby resulting in a slight reduction in Δ*H* [20].

### 3.3. Effects of BGLSP on Thermo-Mechanical Properties of Dough

The impact of BGLSP incorporation on the thermo-mechanical properties of dough is presented in Table 4. An increase in BGLSP concentration from 0% to 10% resulted in an elevation of water absorption from 64.50% to 66.70%. This phenomenon can be attributed to the elevated fiber content in BGLSP, as the hydroxyl groups in fiber can form hydrogen bonds with water molecules, thereby enhancing water absorption [12]. Development time, defined as the duration required for flour dough to achieve its maximum consistency post-mixing [22], increased from 3.32 min to 5.96 min with the addition of BGLSP. Sui et al. [23] demonstrated that an increased proportion of wheat bran in formulated flour is associated with a decrease in gluten content, which in turn results in prolonged development time and enhanced dough stability. Consequently, an extended duration for gluten network formation led to a prolonged dough development time. Dough stability time, indicative of the dough’s mechanical resistance, was significantly enhanced (*p* < 0.05) with the addition of BGLSP compared to the control sample. This enhancement is likely due to the dispersion of fiber within the dough, which increases kneading resistance owing to its inherent rigidity [12]. Furthermore, an increase in dough stability time was observed for flours with incorporated BGLSP, attributed to gluten dilution, as BGLSP is a non-gluten flour [10]. Table 4 provides the values for C1, representing the maximum torque during the initial mixing phase, and C2, indicating the weakening of the protein network. The C1 values of the dough remained relatively unchanged with the incorporation of BGLSP, whereas the C2 values exhibited a declining trend. The addition of BGLSP results in a less compact protein structure, thereby enhancing the availability of enzymatic attachment sites [24]. Consequently, this facilitates an accelerated rate of protein weakening due to thermal effects, leading to a reduction in C2. In a similar study, Wang et al. [12] observed a gradual decrease in the C2 value from 0.53 to 0.35 Nm as the levels of insoluble dietary fiber and ferulic acid were increased.

### 3.4. Effects of BGLSP on Physical Characteristics of CSB

**Figure 4:**
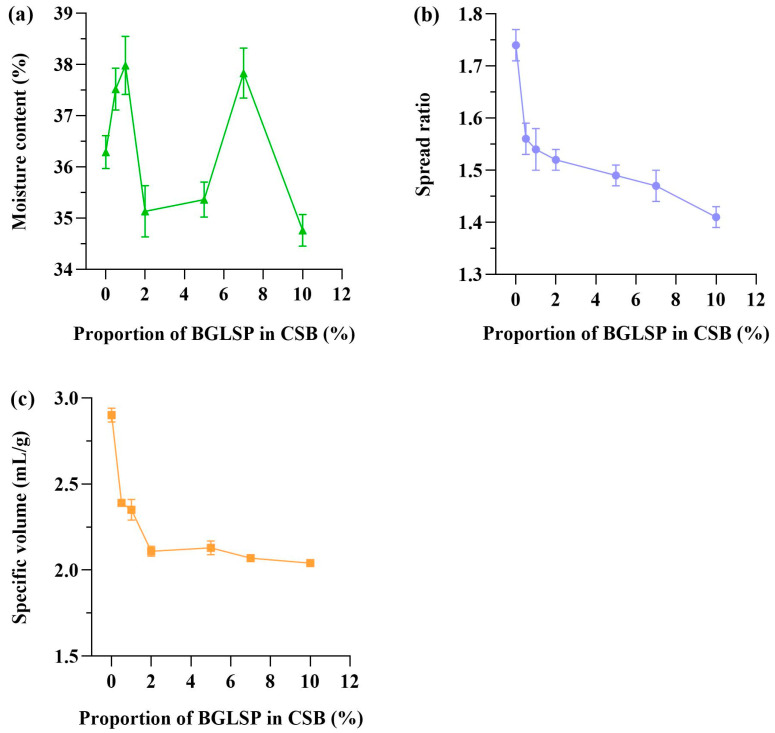
Effects of BGLSP on (**a**) moisture content, (**b**) spread ratio, and (**c**) specific volume of CSB. Abbreviations: BGLSP, broken *G. lucidum* spore powder; CSB, Chinese steamed bread.

Figure 4 illustrates the effects of varying BGLSP substitutions on the moisture content, spread ratio, and specific volume of CSB. The moisture content of CSB is a critical factor influencing its shelf life and susceptibility to deterioration. As depicted in Figure 4a, the moisture content in CSBs exhibited variability. Notably, the moisture levels in CSBs increased significantly as the BGLSP concentration rose from 5% to 7%, likely attributable to the enhanced fiber content (*p* < 0.05) [12]. The spread ratio and specific volume are crucial quality indicators for CSB. An increase in specific volume and a decrease in the height-to-diameter ratio enhance the esthetic appeal of CSB, thereby increasing its consumer preference. Research indicates that the augmented specific volume and improved softness of CSB can be ascribed to the gluten’s capacity to retain air within the dough [25]. Figure 4b,c shows a significant decrease (*p* < 0.05) in these indicators with increased BGLSP levels. At 10% BGLSP, the spread ratio and specific volume decreased by 18.96% and 29.65%, respectively, compared to CSB made from wheat flour. This reduction could be attributed to the enhanced dilution of gluten proteins by BGLSP, coupled with the limited utilization of dietary fiber in BGLSP by yeast [12]. BGLSP could compete with gluten for water absorption, weakening gluten’s air-holding capacity and thus reducing these quality metrics [18].

### 3.5. Effects of BGLSP on Textural Properties of CSB

The textural characteristics of CSB serve as intuitive indicators of its quality and can be quantified using parameters such as hardness, chewiness, gumminess, springiness, and cohesiveness. Table 5 presents the textural properties of CSB with varying levels of BGLSP additions, as determined through textural profile analysis. The inclusion of 10% BGLSP resulted in a significant increase in the hardness, chewiness, and gumminess of CSB (*p* < 0.05). Hardness is a critical parameter influencing the sensory quality of CSB and serves as a primary indicator of aging during storage [26]. BGLSP is rich in fiber, lipids, and protein components, which can interact with gluten and starch, thereby affecting the formation of the gluten network [1]. Similarly, Gao et al. [10] reported that the addition of flaxseed flour led to an increase in the hardness of CSB. Cohesiveness reflects the strength of the internal bonds within the CSB, and lower cohesiveness may be preferred by certain consumers in China [26]. Springiness is typically used to describe the ability of an object to return to its original shape after deformation [27]. As demonstrated in Table 5, the incorporation of BGLSP resulted in a reduction in the cohesiveness and springiness of CSB compared to the control sample. This phenomenon may be attributed to the dilution of gluten protein, which is likely caused by the steric hindrance effects of fiber [12].

### 3.6. Effects of BGLSP on Flavor Characteristics of CSB

**Figure 5:**
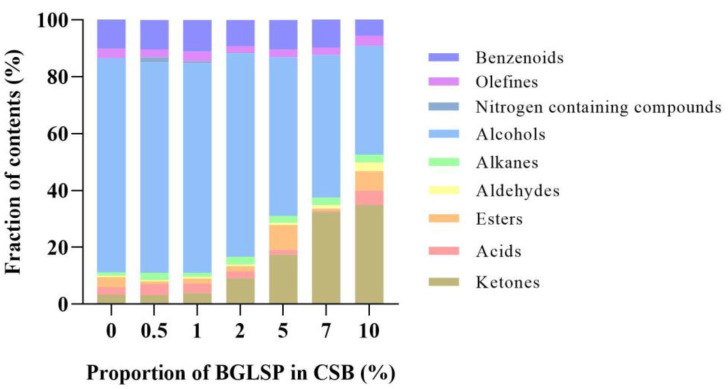
Effects of BGLSP on flavor characteristics of CSB. Abbreviations: BGLSP, broken *G. lucidum* spore powder; CSB, Chinese steamed bread.

GC–MS data of major volatile compounds isolated from CSBs using HS-SPME are summarized in Figure 5 and Table 6. A total of 115 volatile compounds from nine different chemical classes were identified in CSBs. These compounds included 7 ketones, 10 acids, 23 esters, 9 aldehydes, 27 alkanes, 20 alcohols, 1 nitrogen containing compound, 9 olefins, and 9 benzenoids. It is commonly reported that the pathway of flavor compound formation in CSB mainly includes lipid oxidation, heat treatment, and microbial metabolism [10]. Alcohols and benzenoids, which were predominant in the extracts of the control sample (75.36% and 10.11% of the total volatiles, respectively), were the most prominent compounds. The fruity and grassy fragrances detected in the control sample were primarily attributed to the presence of alcohols, which have been previously identified as key odorants in control CSB [28]. As the proportion of BGLSP increased, the content of alcohols decreased, while the content of ketones and aldehydes increased significantly, and both groups reached a maximum when the addition proportion of BGLSP was 10%. Aldehydes and ketones are associated with lipid oxidation, which contributes to the development of fatty, green, nutty, or buttery odors [16]. Monitoring the oxidative degradation of unsaturated fatty acids is essential for ensuring CSB quality and producing healthier, safer, and more appealing foods.

### 3.7. Effects of BGLSP on Sensory Characteristics of CSB

**Figure 6:**
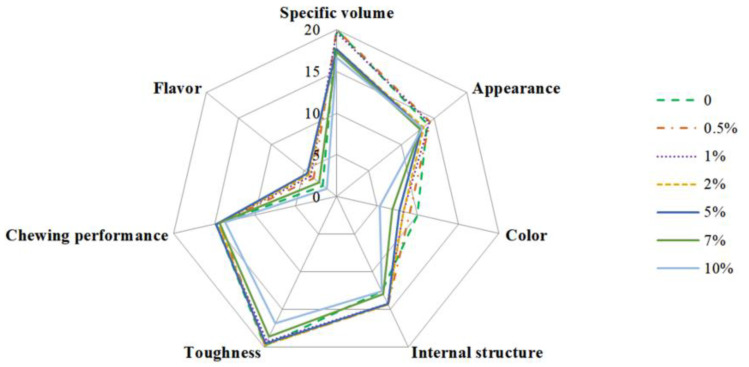
Effects of BGLSP on sensory characteristics of CSB. Abbreviations: BGLSP, broken *G. lucidum* spore powder; CSB, Chinese steamed bread.

Sensory evaluation serves as a crucial metric for assessing food quality, primarily relying on human senses such as sight, smell, and taste to appraise the sensory attributes of food, thereby elucidating consumer preferences for various products [21]. The sensory evaluation outcomes for CSB are presented in Figure 6. As depicted in Figure 6, the inclusion of BGLSP at concentrations ranging from 0.5% to 5% resulted in relatively high flavor scores, indicating that the aroma of BGLSP may enhance the quality of CSB. Conversely, increases in BGLSP concentration from 0.5% to 10% were associated with declines in specific volume, appearance, color, internal structure, and texture scores of CSB. These findings align with the previously determined physicochemical properties of flour blends and the quality characteristics of CSB. Regarding overall acceptability, the sensory evaluation scores for CSB with 0.5% and 1% BGLSP were significantly higher than those of other samples, with scores of 95.37 and 94.30, respectively. Prior research has similarly indicated that the excessive incorporation of flaxseed flour can adversely affect the sensory scores of CSB [1,29].

## 4. Conclusions

This study assessed the impact of BGLSP supplementation on the physicochemical properties of flour blends and dough, as well as the quality of CSB. The incorporation of BGLSP into wheat flour significantly affected the thermal properties of the flour blends and the thermo-mechanical properties of the dough. While BGLSP improved the flavor profile of CSB, excessive addition (ranging from 2% to 10%) negatively influenced the color, spread ratio, specific volume, and texture of the CSB. Future research should explore the pretreatment (e.g., steam explosion) of BGLSP prior to its incorporation, alongside other ingredients, to enhance structural stability and achieve the optimal texture, flavor, and nutritional profile of BGLSP-enriched CSB. Additionally, as the present study exclusively examined the impact of BGLSP on the properties and quality of dough and CSB, the nutrient digestion and absorption characteristics of CSB have not been elucidated. Consequently, our forthcoming research will focus on identifying and assessing the bioavailability of bioactive compounds in CSB using an in vitro digestion model.

## Figures and Tables

**Table 1:** Sensory quality scoring system for CSB.

Quality Parameter	Score	Sensory Assessment Standard
Specific volume (mL/g)	20	Specific volume ≥ 2.3: 20.
		1.7 ≤ Specific volume ≤ 2.3: minus 1 score per 0.1 mL/g reduction.
		Specific volume ≤ 1.7: 3–6.
Appearance	15	Smooth: 12.1–15.
		Uneven, pliable, air bubble, concave or large burns: 1–12.
Color	10	White, milky, milky white: 8.1–10.
		Light yellow, yellow: 6.1–8.
		Gloom, dark: 1–6.
Internal structure	15	Longitudinal section with small pores evenly: 12.1–15.
		Close pores, uniform or separated between the edges and the epidermis: 9.1–12.
		Large pores, rough structure and uneven: 1–9.
Toughness	20	Rebound quickly, can be compressed more than 1/2 after finger press: 16.1–20.
		Rebound slowly or does not rebound after finger press: 12.1–16.
		Difficulty pressing fingers, very hard: 1–12.
Chewing performance	15	Great chewing without teeth stickness: 12.1–15.
		Chewing is not refreshing, slightly sticky or sticky: 1–12.
Flavor	5	Wheat fragrance, no smell: 4.1–5.
		Bland flavor: 3.1–4.
		Unpleasant smell: 1–3.

Note: Scoring system: poor ≤ 70, average or general or acceptable = 70–79, good = 80–89, excellent = 90–100. Abbreviations: CSB, Chinese steamed bread.

**Table 2:** Effects of BGLSP on color of flour blends.

Addition Amount (%)	L*	a*	b*	Δ*E*
0	97.74 ± 0.14 a	−0.07 ± 0.04 f	15.29 ± 0.06 b	−
0.5	91.42 ± 0.09 b	1.16 ± 0.06 e	13.32 ± 0.03 d	6.73 ± 0.20 f
1	89.39 ± 0.20 c	1.28 ± 0.09 e	12.65 ± 0.08 d	8.86 ± 0.29 e
2	80.25 ± 0.59 d	3.24 ± 0.21 d	13.02 ± 0.23 d	17.94 ± 0.45 d
5	68.94 ± 0.54 e	5.22 ± 0.20 c	14.16 ± 0.42 c	29.30 ± 0.41 c
7	60.79 ± 0.14 f	7.29 ± 0.04 b	16.59 ± 0.16 a	37.69 ± 0.29 b
10	55.19 ± 0.31 g	8.04 ± 0.21 a	16.59 ± 0.43 a	43.34 ± 0.49 a

Results are means ± SD of triplicate determinations. Column values with different lowercased letters are significantly different (*p* < 0.05); “−”, non-detectable. Abbreviations: BGLSP, broken *G. lucidum* spore powder.

**Table 3:** Effects of BGLSP on thermal properties of flour blends.

Addition Amount (%)	Temperature (°C)			Δ*H* (J/g)
	*T*_o_	*T*_p_	*T*_c_	
0	56.88 ± 0.10 b	62.62 ± 0.14 ab	68.27 ± 0.31 ab	6.99 ± 0.53 ab
0.5	56.94 ± 0.01 b	62.43 ± 0.05 b	67.39 ± 0.05 b	5.89 ± 0.01 b
1	56.87 ± 0.11 b	62.88 ± 0.02 ab	67.98 ± 0.07 ab	5.93 ± 0.33 b
2	56.85 ± 0.05 b	63.01 ± 0.59 ab	68.71 ± 0.81 a	7.24 ± 0.48 a
5	56.88 ± 0.03 b	62.61 ± 0.00 ab	67.46 ± 0.00 b	6.64 ± 0.24 ab
7	57.59 ± 0.01 a	63.04 ± 0.00 ab	67.60 ± 0.08 ab	5.86 ± 0.00 b
10	57.40 ± 0.02 a	63.29 ± 0.07 a	68.57 ± 0.00 ab	6.25 ± 0.16 ab

Results are means ± SD of triplicate determinations. Column values with different lowercased letters are significantly different (*p* < 0.05). Abbreviations: BGLSP, broken *G. lucidum* spore powder; *T*_o_, onset temperature; *T*_p_, peak temperature; *T*_c_, conclusion temperature; Δ*H*, enthalpy change.

**Table 4:** Effects of BGLSP on thermo-mechanical properties of dough.

Addition Amount (%)	Water Absorption (min)	Development Time (min)	Stability Time (min)	C1 (Nm)	C2 (Nm)
0	64.50 ± 0.00 f	3.32 ± 0.00 f	1.56 ± 0.06 e	1.06 ± 0.00 f	0.29 ± 0.01 b
0.5	65.20 ± 0.00 e	4.15 ± 0.10 d	2.02 ± 0.06 d	1.08 ± 0.00 e	0.27 ± 0.00 bc
1	65.45 ± 0.15 d	3.63 ± 0.00 e	2.13 ± 0.03 d	1.20 ± 0.01 a	0.30 ± 0.00 a
2	65.50 ± 0.00 d	3.50 ± 0.04 e	2.50 ± 0.01 c	1.15 ± 0.00 c	0.30 ± 0.02 a
5	66.10 ± 0.10 c	4.91 ± 0.01 c	3.44 ± 0.02 b	1.17 ± 0.00 b	0.27 ± 0.00 bc
7	66.40 ± 0.00 b	5.25 ± 0.07 b	3.38 ± 0.01 b	1.19 ± 0.01 a	0.27 ± 0.01 bc
10	66.70 ± 0.00 a	5.96 ± 0.02 a	5.57 ± 0.05 a	1.12 ± 0.00 d	0.24 ± 0.00 c

Results are means ± SD of triplicate determinations. Column values with different lowercased letters are significantly different (*p* < 0.05). Abbreviations: BGLSP, broken *G. lucidum* spore powder; C1, initial consistency; C2, protein network weakening.

**Table 5:** Effects of BGLSP on textural properties of CSB.

Addition Amount (%)	Hardness (g)	Chewiness (mJ)	Gumminess (g)	Springiness (mm)	Cohesiveness
0	1980.30 ± 37.33 c	1890.05 ± 41.90 b	1809.13 ± 42.70 b	7.34 ± 0.07 a	1.04 ± 0.01 a
0.5	1947.67 ± 36.81 c	1877.12 ± 86.38 b	1833.70 ± 88.06 b	7.57 ± 0.08 a	0.95 ± 0.04 ab
1	1966.19 ± 26.39 c	1836.59 ± 92.79 b	1858.61 ± 63.57 b	7.30 ± 0.01 a	0.95 ± 0.06 ab
2	1985.22 ± 17.91 c	1869.43 ± 36.65 b	1834.95 ± 57.37 b	7.46 ± 0.04 a	0.88 ± 0.01 ab
5	2289.90 ± 121.39 b	1853.89 ± 57.17 b	1933.25 ± 33.04 b	7.24 ± 0.05 ab	0.85 ± 0.02 b
7	2371.52 ± 90.08 b	1908.32 ± 72.37 b	1964.54 ± 48.60 b	6.85 ± 0.27 bc	0.79 ± 0.09 b
10	2911.38 ± 89.78 a	2294.41 ± 30.16 a	2381.66 ± 43.73 a	6.63 ± 0.11 c	0.79 ± 0.05 b

Results are means ± SD of triplicate determinations. Column values with different lowercased letters are significantly different (*p* < 0.05). Abbreviations: BGLSP, broken *G. lucidum* spore powder; CSB, Chinese steamed bread.

**Table 6:** Volatile compounds identified in CSBs prepared in different flour blends.

Addition Amount (%)	Volatile Components (%)								
	Ketones	Acids	Esters	Aldehydes	Alkanes	Alcohols	Nitrogen Containing Compounds	Olefines	Benzenoids
0	3.36 ± 0.02 f	2.70 ± 0.03 d	3.50 ± 0.01 c	0.49 ± 0.01 e	1.15 ± 0.04 d	75.36 ± 0.72 a	0.00 ± 0.00 e	3.33 ± 0.06 b	10.11 ± 0.08 d
0.5	3.21 ± 0.00 f	3.85 ± 0.01 b	0.99 ± 0.00 e	0.46 ± 0.01 e	2.53 ± 0.02 b	73.97 ± 0.69 a	1.87 ± 0.02 a	2.79 ± 0.08 c	10.34 ± 0.04 c
1	3.95 ± 0.01 e	3.26 ± 0.01 c	1.83 ± 0.01 d	0.63 ± 0.00 d	1.38 ± 0.03 c	73.69 ± 0.77 a	0.83 ± 0.01 b	3.33 ± 0.03 b	11.11 ± 0.02 b
2	9.05 ± 0.05 d	2.51 ± 0.03 e	1.81 ± 0.03 d	0.62 ± 0.01 d	2.74 ± 0.02 a	71.44 ± 0.16 b	0.30 ± 0.01 c	2.30 ± 0.04 e	9.22 ± 0.07 f
5	17.48 ± 0.13 c	1.62 ± 0.00 f	8.77 ± 0.05 a	0.75 ± 0.03 c	2.45 ± 0.02 b	55.82 ± 0.32 c	0.06 ± 0.00 d	2.59 ± 0.03 d	10.48 ± 0.04 c
7	32.50 ± 0.11 b	0.41 ± 0.00 g	0.74 ± 0.02 f	1.26 ± 0.05 b	2.54 ± 0.01 b	50.26 ± 0.28 d	0.02 ± 0.00 e	2.56 ± 0.01 d	9.72 ± 0.08 e
10	34.86 ± 0.28 a	5.11 ± 0.02 a	6.80 ± 0.07 b	3.07 ± 0.03 a	2.77 ± 0.03 a	38.22 ± 0.14 e	0.00 ± 0.00 e	3.62 ± 0.06 a	11.66 ± 0.06 a

Results are means ± SD of triplicate determinations. Column values with different lowercased letters are significantly different (*p* < 0.05). Abbreviations: CSB, Chinese steamed bread.

## Data Availability

The original contributions presented in this study are included in the article. Further inquiries can be directed to the corresponding authors.
